# Clinical significance of serum glucose to lymphocyte ratio as a prognostic marker in peritoneal dialysis patients

**DOI:** 10.1080/0886022X.2023.2224893

**Published:** 2023-06-19

**Authors:** Jiexin Chen, Ruiying Tang, Xiaojiang Zhan, Jihong Deng, Yanxia Zhang, Haibo Long, Fenfen Peng, Na Tian, Yueqiang Wen, Xiaoyang Wang, Xiaoran Feng, Ning Su, Xingming Tang, Xianfeng Wu, Qian Zhou, Qingdong Xu

**Affiliations:** a Department of Nephrology, Jiangmen Central Hospital, Jiangmen, China; b Department of Nephrology, The First Affiliated Hospital of Nanchang University, Nanchang, China; c Department of Nephrology, Zhujiang Hospital, Southern Medical University, Guangzhou, China; d Department of Nephrology, General Hospital of Ningxia Medical University, Yinchuan, China; e Department of Nephrology, The Second Affiliated Hospital of Guangzhou Medical University, Guangzhou, China; f Department of Nephrology, The First Affiliated Hospital of Zhengzhou University, Zhengzhou, China; g Department of Nephrology, Jiujiang NO.1 people’s Hospital, Jiujiang, China; h Department of Hematology, The Sixth Affiliated Hospital of Sun Yat-Sen University, Guangzhou, China; i Department of Nephrology, DongGuan SongShan Lake Tungwah Hospital, DongGuan, China; j Department of Nephrology, Affiliated Sixth People’s Hospital, Shanghai Jiao Tong Univeristy, Shanghai, China; k Department of Medical Statistics, Clinical Trials Unit, The First Affiliated Hospital, Sun Yat-sen Univeristy, Guangzhou, China

**Keywords:** Peritoneal dialysis, glucose to lymphocyte ratio, all-cause mortality, cardiovascular mortality

## Abstract

**Background:**

The glucose-to-lymphocyte ratio (GLR), a glucose metabolism and systemic inflammatory response parameter, is associated with an adverse prognosis for various diseases. However, the association between serum GLR and prognosis in patients undergoing peritoneal dialysis (PD) is poorly understood.

**Methods:**

In this multi-center cohort study, 3236 PD patients were consecutively enrolled between 1 January 2009 and 31 December 2018. Patients were divided into four groups according to the quartiles of baseline GLR levels (Q1: GLR ≤ 2.91, Q2:2.91 < GLR ≤ 3.91, Q3:3.91 < GLR < 5.59 and Q4: GLR ≥ 5.59). The primary endpoint was all-cause and cardiovascular disease (CVD) related mortality. The correlation between GLR and mortality was examined using Kaplan–Meier and multivariable Cox proportional analyses.

**Results:**

During the follow-up period of 45.93 ± 29.01 months, 25.53% (826/3236) patients died, of whom 31% (254/826) were in Q4 (GLR ≥ 5.59). Multivariable analysis revealed that GLR was significantly associated with all-cause mortality (adjusted HR 1.02; CI 1.00 ∼ 1.04, *p* = .019) and CVD mortality (adjusted HR 1.02; CI 1.00 ∼ 1.04, *p* = .04). Compared with the Q1 (GLR ≤ 2.91), placement in Q4 was associated with an increased risk of all-cause mortality (adjusted HR: 1.26, 95% CI: 1.02 ∼ 1.56, *p* = .03) and CVD mortality (adjusted HR 1.76; CI 1.31 ∼ 2.38, *p* < .001). A nonlinear relationship was found between GLR and all-cause or CVD mortality in patients undergoing PD (*p* = .032).

**Conclusion:**

A higher serum GLR level is an independent prognostic factor for all-cause and CVD mortality in patients undergoing PD, suggesting that more attention should be paid to GLR.

## 1. Introduction

Peritoneal dialysis (PD) is an efficient therapy for renal replacement, and the number of patients managed with dialysis is continuously growing [1]. Current studies suggest that more than 272,000 patients receive PD worldwide, accounting approximately 11% of the global dialysis patients [1]. Due to the global prevalence of COVID-19, PD has gained popularity in recent years owing to its operability at home. However, the mortality rate in patients with PD is high, with cardiovascular diseases accounting for almost 60% of all-cause mortality cases. Because of the increasing number and mortality rate of patients undergoing PD, it is particularly important to identify effective and controllable factors that can predict the outcomes of patients undergoing PD.

Abnormal glucose metabolism and inflammatory properties are associated with poor prognosis in patients undergoing PD [2,3]. In recent decades, increasing attention has been paid to the clinical value of elevated fasting blood glucose, metabolic syndrome, and biomarkers of inflammation in patients with PD. The glucose to lymphocyte ratio (GLR) is a parameter influenced by both glucose metabolism and systemic immune status associated with cancer invasiveness [4] and has been explored in patients with various other diseases. A previous study by Navarro et al. demonstrated that GLR was an independent predictor of both overall and disease-free survival in gallbladder cancer [5]. Moreover, there is a nonlinear relationship between GLR and in-hospital mortality in intensive care patients with sepsis [6]. As an easily available biomarker, GLR can also be used independently to predict in-hospital mortality in patients with acute exacerbation of chronic obstructive pulmonary disease [7]. Another study found that the baseline GLR is an independent prognostic factor in patients with pancreatic cancer, with better sensitivity and specificity than the neutrophil to lymphocyte ratio (NLR), platelet to lymphocyte ratio (PLR), and lymphocyte to monocyte ratio (LMR) [8], which are widely used systemic inflammatory response markers.

However, the association between the GLR and all-cause mortality in patients undergoing PD has rarely been investigated. We performed this multi-center cohort study to address this knowledge gap and test the hypothesis that GLR is associated with all-cause mortality in patients undergoing PD.

## 2. Methods

### 2.1. Patients

**Figure 1.**
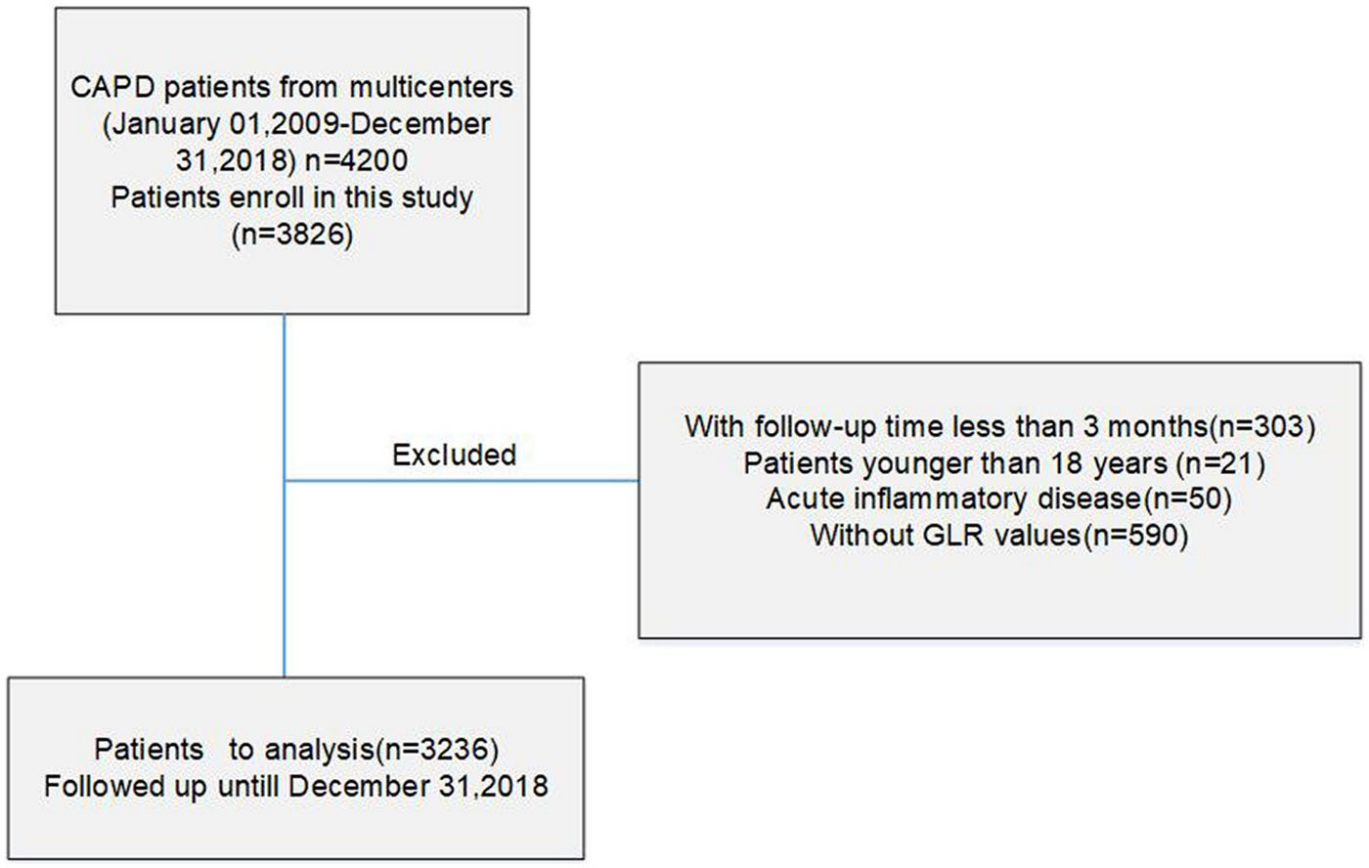
Flow diagram of the study.

In this cohort study, 4200 patients receiving PD at eight different hospitals in China from 01 January 2009 to 31 December 2018, were enrolled and followed-up. The exclusion criteria were as follows: patients aged <18 years at the start of PD (*n* = 21); those who received PD therapy for less than 3 months (*n* = 303); those with acute inflammatory disease, including diseases that may cause high fasting blood glucose (*n* = 50); those lacking baseline GLR values (*n* = 590). Finally, 3236 patients were included in the study and followed up until endpoint or 31 December 2018 (Figure 1). This study was performed in accordance with the ethical standards of the Helsinki Declaration and its later amendments and was approved by the Human Ethics Committees of each study organization.

### 2.2. Clinical variables

Baseline demographic data included sex, age, history of diabetes mellitus (DM), cardiovascular disease (CVD), current smoking and drinking habits, and medication use (including calcium channel blocker, angiotensin-converting enzyme inhibitor, angiotensin receptor blocker, β-blocker, ɑ-blocker, diuretic, Aspirin and Insulin). Clinical and biochemical data included body mass index (BMI), systolic pressure, diastolic pressure, hemoglobin (HB), white blood cell (WBC) count, blood platelet count (PLT), neutrophil count, monocyte count, lymphocyte count, serum albumin (ALB) level, serum creatinine, uric acid (UA), fasting blood glucose, total cholesterol (TC), triglyceride (TG), serum calcium, serum phosphorus, serum intact parathyroid hormone (iPTH), C-reactive protein (CRP) levels, estimated glomerular (eGFR) and K dialyzer clearance of urea (Kt/V). The GLR ratio was calculated as the fasting blood glucose level divided by the lymphocyte level. All baseline data were obtained during the first 3 months of PD.

### 2.3. Follow-up

All patients were followed up until death, including all-cause and CVD mortality. CVD mortality was defined as death caused by heart disease (ischemic heart disease, hypertensive heart disease, rheumatic heart disease, and other heart diseases) or cerebrovascular diseases (hemorrhagic stroke, ischemic stroke, unspecific stroke, sequelae of stroke, hypertensive encephalopathy, and other cerebrovascular [9,10]. The other endpoints were cessation of PD (transfer to hemodialysis, receiving renal transplantation), loss to follow-up, or the end of follow-up (31 December 2018).

### 2.4. Statistical analyses

Data are expressed as the mean ± standard deviation (SD), percentages, or median (25–75% interquartile range). Continuous variables were compared using analysis of variance or the Kruskal–Wallis test, and categorical variables were tested using the *χ*^2^ test or Fisher’s exact test. Patients were divided into four groups based on the quartile values of the GLR. Kaplan–Meier curve was used to compare survival between different groups. Univariate and multivariate Cox proportional hazards regression models and subdistribution hazards model for competing risk analysis were applied to examine associations and identify independent prognostic factors influencing the outcomes. In subgroup analyses, groups were stratified by age (<65 or ≥65 years old), sex (male or female), BMI (<18.5, or ≥18.5 kg/m^2^), history of DM (with or without), and history of CVD (with or without), after adjusting for age, sex, BMI, systolic pressure, diastolic pressure, DM, history of CVD, smoking, alcohol consumption, medication, PLT, HB, UA, ALB, TC, TG, calcium, phosphorus, iPTH, CRP, eGFR and Kt/V. The interaction p-value corresponds to the interaction test between the GLR and subgroup variable of interest. The results are presented as the hazard ratios (HRs) and reported with 95% confidence intervals (CI). Statistical analyses were conducted using Statistical Package Social Science Vision 26.0 (IBM SPSS 26.0) and Empower(R) (www.empowerstats.com, X&Y solutions, Inc. Boston MA). *p* values < .05 were considered significant.

## 3. Results

### 3.1. Baseline characteristics and Correlations between GLR level and Clinical Parameters

A total of 3236 patients were enrolled in the study and followed up for 45.93 ± 29.01 months. Among them, 44.5% were female, and 34.2% had diabetes mellitus. Patients were divided into four groups according to the quartiles of baseline GLR values (Q1: GLR ≤ 2.91, *n* = 800); Q2: 2.91 < GLR ≤ 3.91, *n* = 808; Q3:3.91 < GLR <5.59, *n* = 815; and Q4: GLR ≥ 5.59, *n* = 813). Compared with the reference group (Q1), patients in the group with the highest baseline GLR level (Q4) tended to be older and had higher percentages of DM, but lower WBC, PLT, lymphocyte, and ALB counts (Table 1, all *p* < .05).

**Table 1.** Baseline characteristics of the study participants stratified by GLR quartile.

Variables	Total (*n* = 3236)	Q1 ≤2.91 (*n* = 800)	Q2 2.92–3.91 (*n* = 808)	Q3 3.92–5.58 (*n* = 815)	Q4 ≥5.59 (*n* = 813)	*p* value
Sex, female (%)	1439 (44.5)	381 (47.6)	377 (46.7)	334 (41)	347 (42.7)	.02*
Age (years)	52.6 ± 14.8	49.3 ± 15.0	51.8 ± 14.8	52.9 ± 14.4	56.1 ± 14.1	<.001
BMI (kg/m^2^)	22.3 ± 3.4	22.4 ± 3.5	22.4 ± 3.4	22.2 ± 3.4	22.1 ± 3.4	.243
Smoking, *n* (%)	279 (8.6)	55 (6.9)	70 (8.7)	75 (9.2)	79 (9.7)	.196
Alcohol consumption, *n* (%)	76 (2.3)	17 (2.1)	19 (2.4)	23 (2.8)	17 (2.1)	.751
Diabetes, *n* (%)	1108 (34.2)	225 (28.1)	243 (30.1)	287 (35.2)	353 (43.4)	<.001*
Systolic pressure (mmHg)	146.2 ± 23.7	144.3 ± 24.1	146.2 ± 23.8	146.6 ± 23.6	147.5 ± 23.3	.059
Diastolic pressure (mmHg)	85.2 ± 15.3	85.3 ± 15.1	85.9 ± 15.2	85.8 ± 15.3	83.8 ± 15.5	.025*
CVD, *n* (%)	952 (29.4)	247 (30.9)	242 (30)	228 (28)	235 (28.9)	.605
Medication						
CCB, *n* (%)	2034 (84.0)	501 (85.9)	489 (80.3)	518 (84.9)	526 (84.8)	.036*
ACEI, *n* (%)	784 (24.2)	190 (23.8)	208 (25.7)	194 (23.8)	192 (23.6)	.72
ARB, *n* (%)	940 (29)	240 (30)	259 (32.1)	227 (27.9)	214 (26.3)	.06*
β-blocker, *n* (%)	1116 (50.6)	261 (48.9)	280 (49.9)	298 (52.9)	277 (50.5)	.579
ɑ-blocker, *n* (%)	671 (20.7)	168 (21)	177 (21.9)	162 (19.9)	164 (20.2)	.746
Diuretic	176 (5.4)	44 (5.5)	45 (5.6)	50 (6.1)	37 (4.6)	.563
Aspirin, *n* (%)	236 (13.0)	51 (11.9)	68 (14.6)	59 (12.9)	58 (12.7)	.682
Insulin, *n* (%)	404 (20.7)	96 (20.3)	109 (21.5)	114 (23.8)	85 (17.4)	.099
Laboratory variables						
White blood cell (10^9^/L)	6.8 ± 2.3	7.7 ± 2.2	6.8 ± 2.2	6.3 ± 2.2	6.4 ± 2.5	<.001*
Platelet (10^9^/L)	203.9 ± 70.1	218.9 ± 63.6	206.5 ± 64.6	196.2 ± 74.9	194.473.9	<.001*
Hemoglobin (g/L)	95.6 ± 21.8	100.7 ± 21.5	97.3 ± 22.3	92.6 ± 20.9	91.8 ± 21.3	<.001*
Neutrophils (10^9^/L)	4.6 ± 2.0	4.8 ± 1.9	4.5 ± 2.0	4.4 ± 1.9	4.7 ± 2.2	<.001*
Monocytes (10^9^/L)	0.5 ± 0.3	0.6 ± 0.3	0.5 ± 0.3	0.5 ± 0.3	0.5 ± 0.3	<.001*
Lymphocytes (10^9^/L)	1.3 ± 0.5	1.9 ± 0.4	1.4 ± 0.3	1.1 ± 0.3	0.9 ± 0.4	<.001*
GLR	4.9 ± 3.7	2.4 ± 0.4	3.4 ± 0.3	4.6 ± 0.5	9.1 ± 5.3	<.001*
Albumin (g/L)	34.8 ± 5.6	35.6 ± 5.9	35.1 ± 5.4	34.6 ± 5.5	34.0 ± 5.5	<.001*
Serum creatinine (μmol/L)	810.7 ± 317.4	838.5 ± 314.5	833.6 ± 314.2	809.3 ± 304.1	761.3 ± 330.9	<.001*
Uric acid (mmol/L)	416.5 ± 113.7	412.9 ± 108.4	418.9 ± 112.2	416.5 ± 110.6	417.9 ± 123.2	.728
Glucose (mmol/L)	5.4 ± 2.1	4.4 ± 0.7	4.8 ± 0.9	5.2 ± 1.4	7.2 ± 3.3	<.001*
Cholesterol (mmol/L)	4.6 ± 1.3	4.7 ± 1.4	4.7 ± 1.2	4.6 ± 1.3	4.6 ± 1.4	.03*
Triglyceride (mmol/L)	1.7 ± 1.4	1.7 ± 1.3	1.7 ± 1.2	1.6 ± 1.4	1.7 ± 1.5	.226
Calcium (mmol/L)	2.1 ± 0.3	2.2 ± 0.3	2.1 ± 0.3	2.1 ± 0.3	2.1 ± 0.3	<.001*
Phosphorus (mmol/L)	1.7 ± 0.6	1.7 ± 0.6	1.6 ± 0.5	1.7 ± 0.6	1.7 ± 0.6	.326
Intact Parathyroid hormone (pg/mL)	326.3 ± 258.9	341.2 ± 277.4	318.2 ± 243.2	319.0 ± 248.7	326.8 ± 265.1	.254
CRP	9.0 ± 14.0	8.5 ± 13.0	9.5 ± 15.0	8.7 ± 13.7	9.4 ± 14.3	.375
PLR	176.7 ± 115.2	117.1 ± 38.6	148.8 ± 49.4	179.6 ± 74.9	260.2 ± 179.1	<.001*
NLR	4.1 ± 3.2	2.6 ± 1.1	3.2 ± 1.5	4.0 ± 2.0	6.4 ± 5.1	<.001*
LMR	3.4 ± 4.1	4.4 ± 4.9	3.5 ± 2.9	3.1 ± 3.2	2.6 ± 5.0	<.001*
eGFR (mL/min/1.73 m^2^)	6.8 ± 3.1	6.5 ± 2.9	6.5 ± 2.9	6.8 ± 3.0	7.4 ± 3.5	<.001*
Kt/V	2.2 ± 0.8	2.2 ± 0.7	2.2 ± 0.7	2.2 ± 1.0	2.0 ± 0.7	.342

Continuous variables are shown as the mean ± SD or median (IQR). BMI: body mass index; CVD: cardiovascular disease; CCB: calcium channel blocker; ACEI: angiotensin-converting enzyme inhibitor; ARB: angiotensin receptor blocker; GLR: glucose to lymphocyte ratio; CRP: C-reactive protein; PLR: platelet/lymphocyte ratio; NLR: neutrophil-to-lymphocyte ratio; LMR: lymphocyte/monocyte ratio; eGFR: estimated glomerular; Kt/V: K dialyzer clearance of urea.

**Figure 2.**
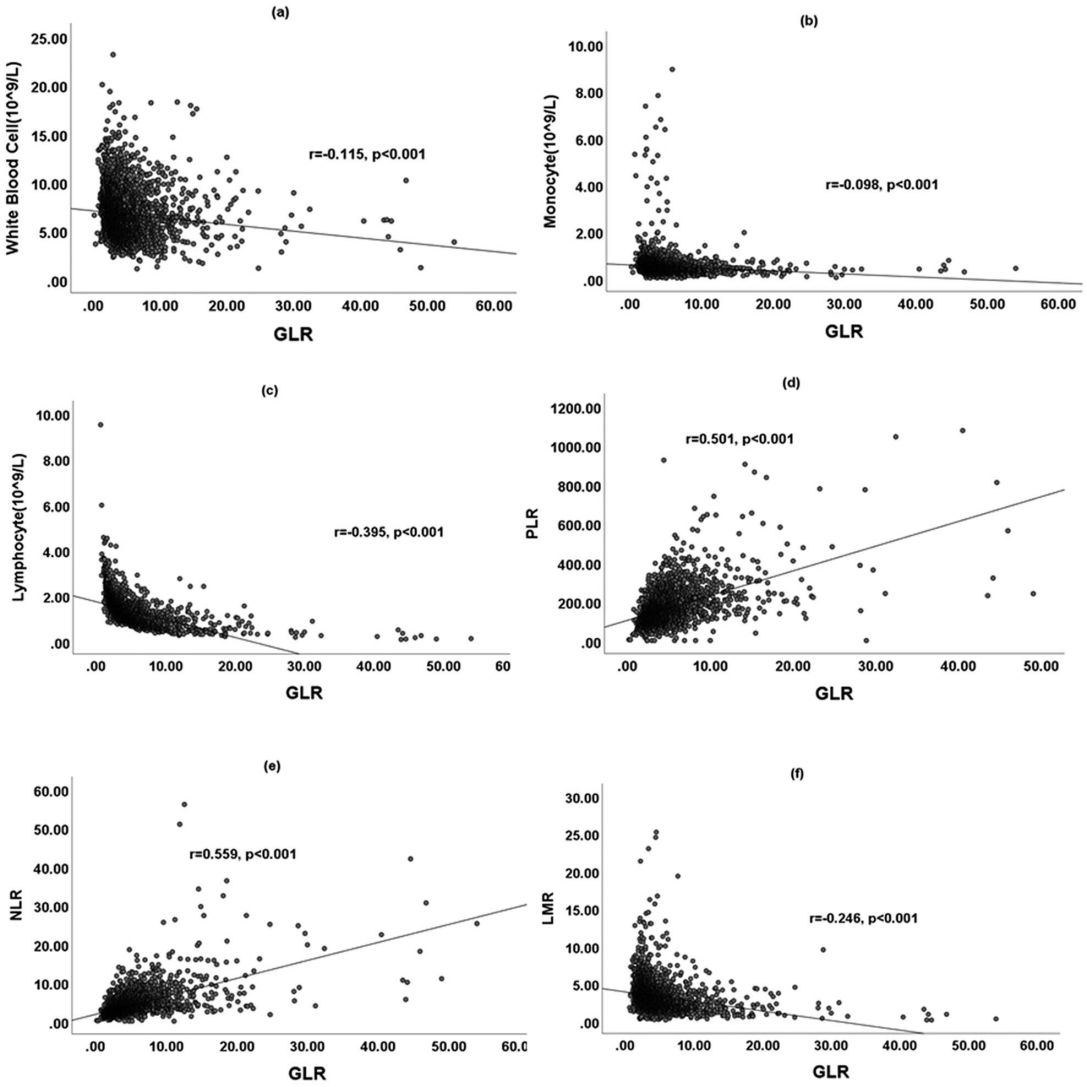
Correlations between GLR level and Clinical Parameters. PLR: platelet/lymphocyte ratio; NLR: neutrophil-to-lymphocyte ratio; LMR: lymphocyte/monocyte ratio.

Spearman’s analyses revealed that the GLR level was positively correlated with PLR and NLR, but negatively correlated with WBC, monocyte, lymphocyte, and LMR (all *p* < .05, Figure 2).

### 3.2. Effect of GLR on all-cause mortality in patients undergoing PD

**Figure 3.**
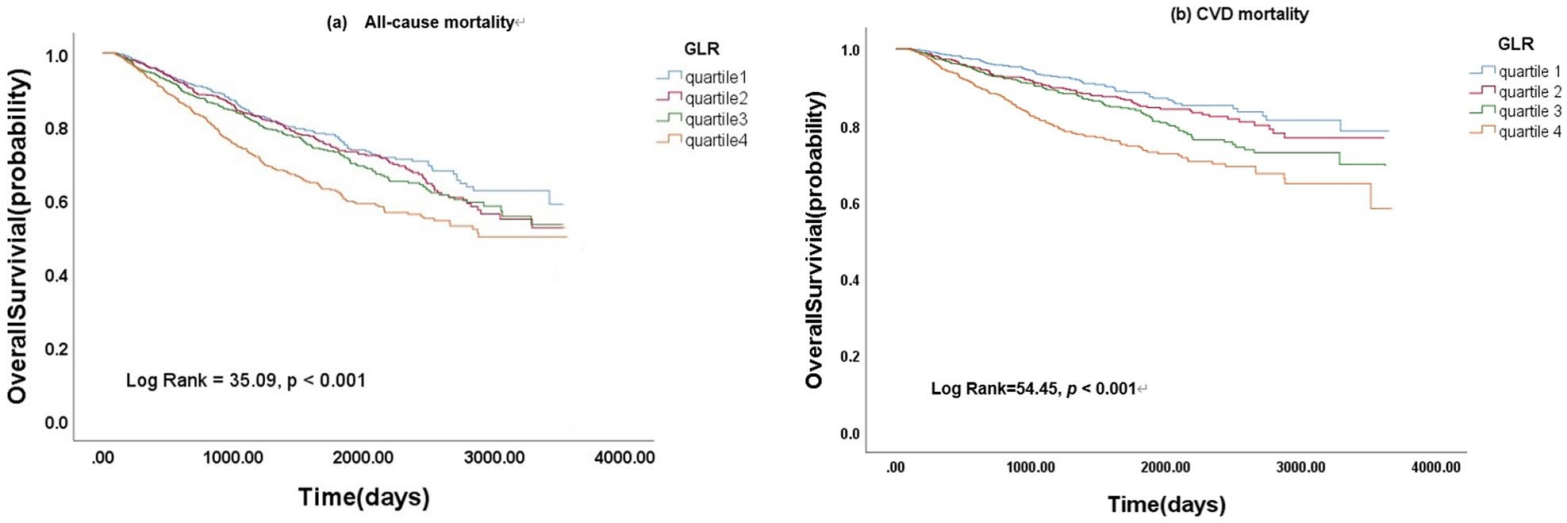
Kaplan-Meier curves of all-cause and CVD mortality stratified by GLR.

**Figure 4.**
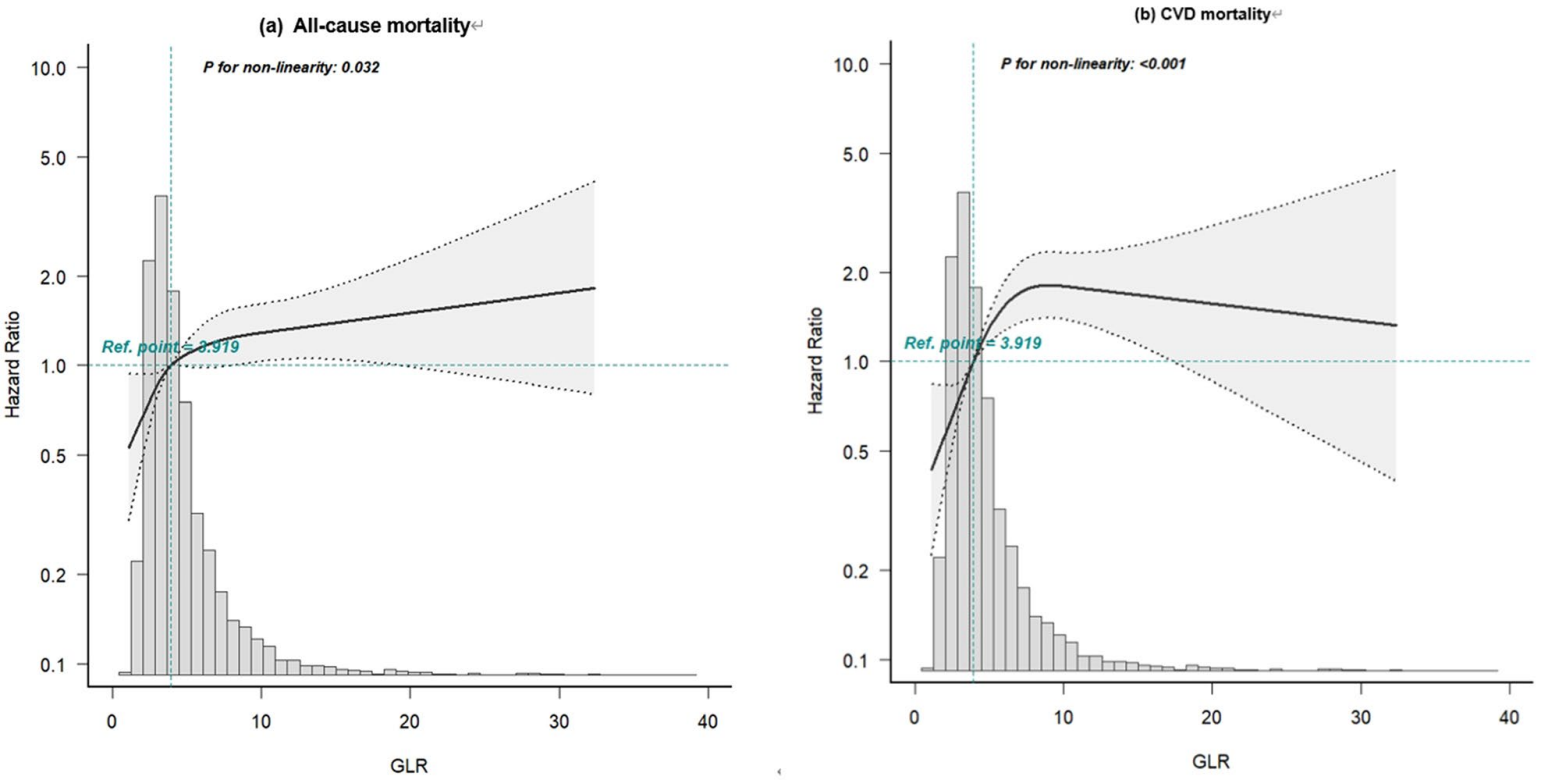
HR and 95% CI for the risk of all-cause and CVD mortality in PD patients, and the changes in GLR from the restricted cubic Spline model.

Overall, 826 participants died in the study period, including 458 (55.4%) who died from CVD. The Kaplan-Meier curves indicated that the highest quartiles of GLR (Q4) were related to shorter overall survival for both all-cause mortality and CVD mortality (Figure 3, *p* < .001). In addition, the restricted cubic splines showed a nonlinear association between GLR and all-cause mortality or CVD mortality (Figure 4, *p* = .032).

### 3.3. Independent prognostic factors for all-cause mortality in patients undergoing PD

The correlation between GLR and all-cause mortality was demonstrated using Cox proportional hazard regression models. Univariate analysis revealed that GLR was a risk factor for all-cause mortality. After fully adjusting for age, sex, BMI, systolic pressure, diastolic pressure, DM, history of CVD, smoking, alcohol consumption, medication, PLT, HB, UA, ALB, TC, TG, calcium, phosphorus, iPTH, CRP, eGFR and Kt/V, multivariable analysis revealed that a higher GLR was independently associated with increased risks of all-cause mortality (HR: 1.02, 95% CI: 1.00–1.04, *p* = .019) and CVD mortality (HR: 1.02, 95% CI: 1.00–1.04, *p* = .040). It is worth noting that, after adjusting for the above-mentioned confounding factors in the similar model, patients with GLR ≥ 5.59 (Q4) carried a 26% (95% CI: 1.02–1.56, *p* = .030) and 76% (95% CI:1.31–2.38, *p* < .001) higher risk of all-cause and CVD mortality, respectively (Table 2).

**Table 2.** Associations of GLR and GLR categories with all-cause mortality and CVD mortality.

Variables	Model 1		Model 2		Model 3	
	HR (95% CI)	*p* value	HR (95% CI)	*p* value	HR (95% CI)	*p* value
All-cause mortality						
GLR^*^	1.03 (1.02–1.05)	<.001	1.02 (1.01–1.04)	.002	1.02 (1.00–1.04)	.019
Q 1	Reference		Reference		Reference	
Q 2	1.13 (0.91–1.39)	.265	1.05 (0.85–1.29)	.662	0.99 (0.80–1.24)	.970
Q 3	1.27 (1.03–1.55)	.024	1.11 (0.9–1.36)	.332	1.01 (0.81–1.25)	.955
Q 4	1.86 (1.53–2.25)	<.001	1.42 (1.17–1.74)	<.001	1.26 (1.02–1.15)	.030
CVD mortality						
GLR	1.04 (1.02–1.05)	<.001	1.02 (1.01–1.04)	.012	1.02 (1.00–1.04)	.040
Q 1	Reference		Reference		Reference	
Q 2	1.3 (0.96–1.76)	.084	1.2 (0.89–1.62)	.237	1.18 (0.86–1.63)	.298
Q 3	1.61 (1.21–2.15)	.001	1.39 (1.04–1.86)	.026	1.30 (0.96–1.78)	.093
Q 4	2.49 (1.9–3.28)	<.001	1.91 (1.44–2.52)	<.001	1.76 (1.31–2.38)	<.001

Cox regression analysis. GLR: glucose to lymphocyte ratio; BMI: body mass index; DM: diabetes; CVD: cardiovascular disease; CCB: calcium channel blocker; ACEI: angiotensin-converting enzyme inhibitor; ARB: angiotensin receptor blocker; PLT: platelet; HB: hemoglobin; UA: Uric acid; ALB: albumin; TC: cholesterol; TG: triglyceride; iPTH: intact Parathyroid hormone; CRP: C-reactive protein; eGFR: estimated glomerular; Kt/V: K dialyzer clearance of urea.

*The two variables in the GLR and GLR categories were included separately in the Cox models.

Model 1: unadjusted.

Model 2: adjusted for age, sex, BMI, systolic pressure, diastolic pressure, DM, and CVD history.

Model 3: Model 2 plus smoking, alcohol consumption, medication (CCB, ACEI, ARB, β-blocker, ɑ-blocker, Diuretic, Aspirin, Insulin), PLT, HB, UA, ALB, TC, TG, calcium, phosphorus, iPTH, CRP, eGFR and Kt/V.

**Figure 5.**
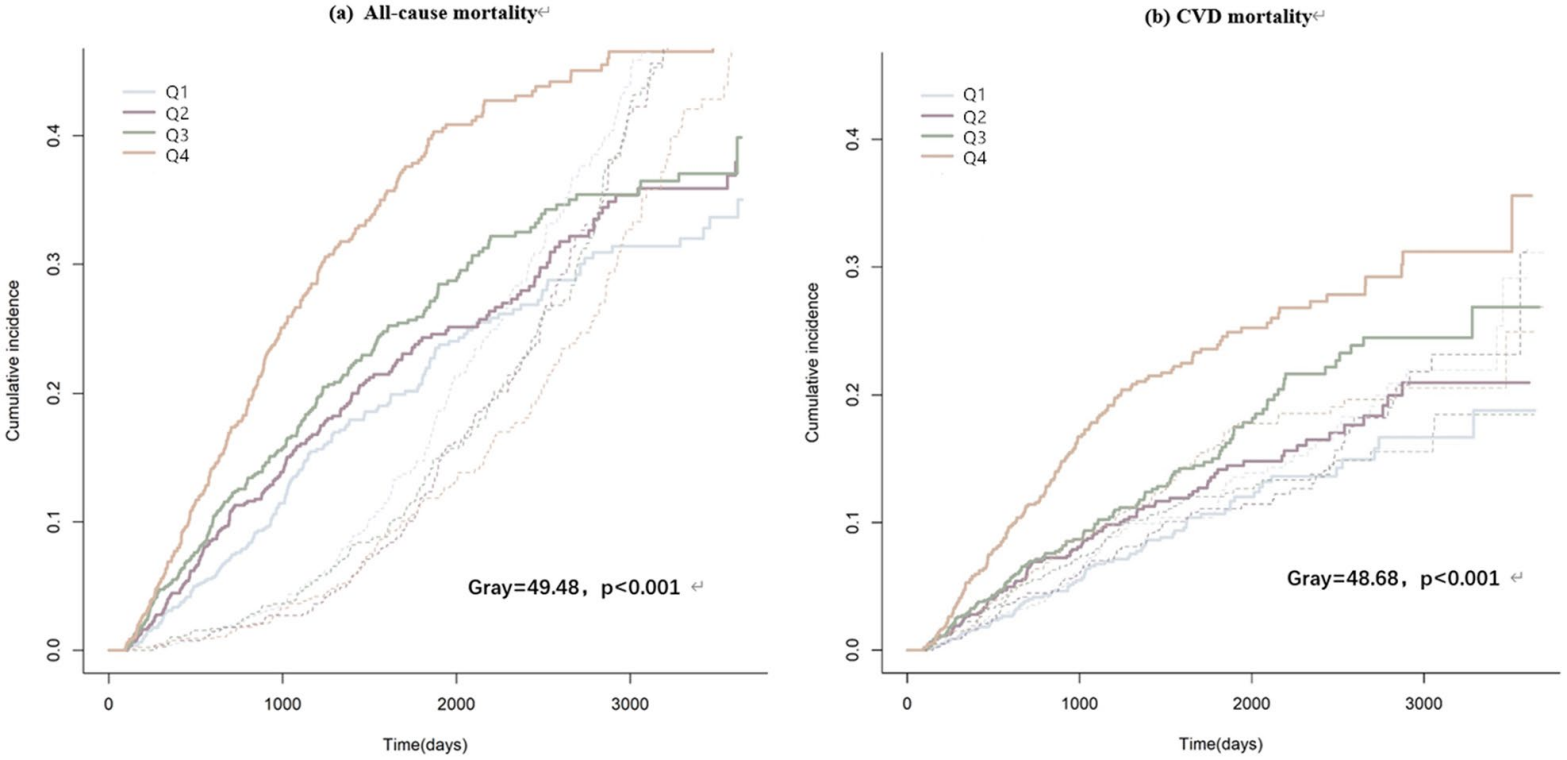
Cumulative event rates curves of all-cause and CVD mortality in different GLR value.

Fine–Gray competing risk model was used for further sensitivity analysis. It showed that high level of GLR was associated with high all-cause mortality (Gray = 49.48, *p* < .001) and CVD mortality (Gray = 48.68, *p* < .001) (Figure 5). After adjusting for multiple factors, compared with Q2 and Q3, patients in the highest GLR group had a 31% (SHR = 1.31, 95% CI: 1.07–1.60, *p* = .008) and 89% (SHR = 1.89, 95% CI: 1.42–2.51, *p* < .001) increased risk of all-cause mortality and CVD mortality, significantly (Table 3).

**Table 3.** Associations of GLR and GLR categories with all-cause mortality and CVD mortality (Fine–Gray competing risk model).

Variables	Model 1		Model 2		Model 3	
	SHR (95% CI)	*p* value	SHR (95% CI)	*p* value	SHR (95% CI)	*p* value
All-cause mortality						
GLR	1.03 (1.02–1.05)	<.001	1.02 (1.00–1.03)	.019	1.02 (1.00–1.03)	.050
Q 1	Reference		Reference		Reference	
Q 2	1.14 (0.93–1.40)	.210	1.04 (0.85–1.27)	.720	0.99 (0.81–1.22)	.950
Q 3	1.27 (1.03–1.55)	.023	1.08 (0.88–1.32)	.450	1.02 (0.83–1.25)	.840
Q 4	1.87 (1.55–2.27)	<.001	1.40 (1.15–1.70)	.001	1.31 (1.07–1.60)	.008
CVD mortality						
GLR	1.03 (1.02–1.05)	<.001	1.02 (1.01–1.04)	.005	1.02 (1.01–1.04)	.011
Q 1	Reference		Reference		Reference	
Q 2	1.30 (0.97–1.76)	.081	1.21 (0.90–1.64)	.210	1.19 (0.88–1.62)	.260
Q 3	1.60 (1.20–2.12)	.001	1.41 (1.06–1.87)	.019	1.38 (1.03–1.84)	.030
Q 4	2.40 (1.83–3.16)	<.001	1.89 (1.44–2.50)	<.001	1.89 (1.42–2.51)	<.001

SHR: Subdistribution Hazard Ratio; GLR: glucose to lymphocyte ratio; BMI: body mass index; DM: diabetes; CVD: cardiovascular disease; CCB: calcium channel blocker; ACEI: angiotensin-converting enzyme inhibitor; ARB: angiotensin receptor blocker; PLT: platelet; HB: hemoglobin; UA: Uric acid; ALB: albumin; TC: cholesterol; TG: triglyceride; iPTH: intact Parathyroid hormone; CRP: C-reactive protein; eGFR: estimated glomerular; Kt/V: K dialyzer clearance of urea.

Model 1: unadjusted.

Model 2: adjusted for age, sex, BMI, systolic pressure, diastolic pressure, DM, and CVD history.

Model 3: Model 2 plus smoking, alcohol consumption, medication (CCB, ACEI, ARB, β-blocker, ɑ-blocker, Diuretic, Aspirin, Insulin), PLT, HB, UA, ALB, TC, TG, calcium, phosphorus, iPTH, CRP, eGFR and Kt/V.

### 3.4. Subgroups and interaction analyses

**Figure 6.**
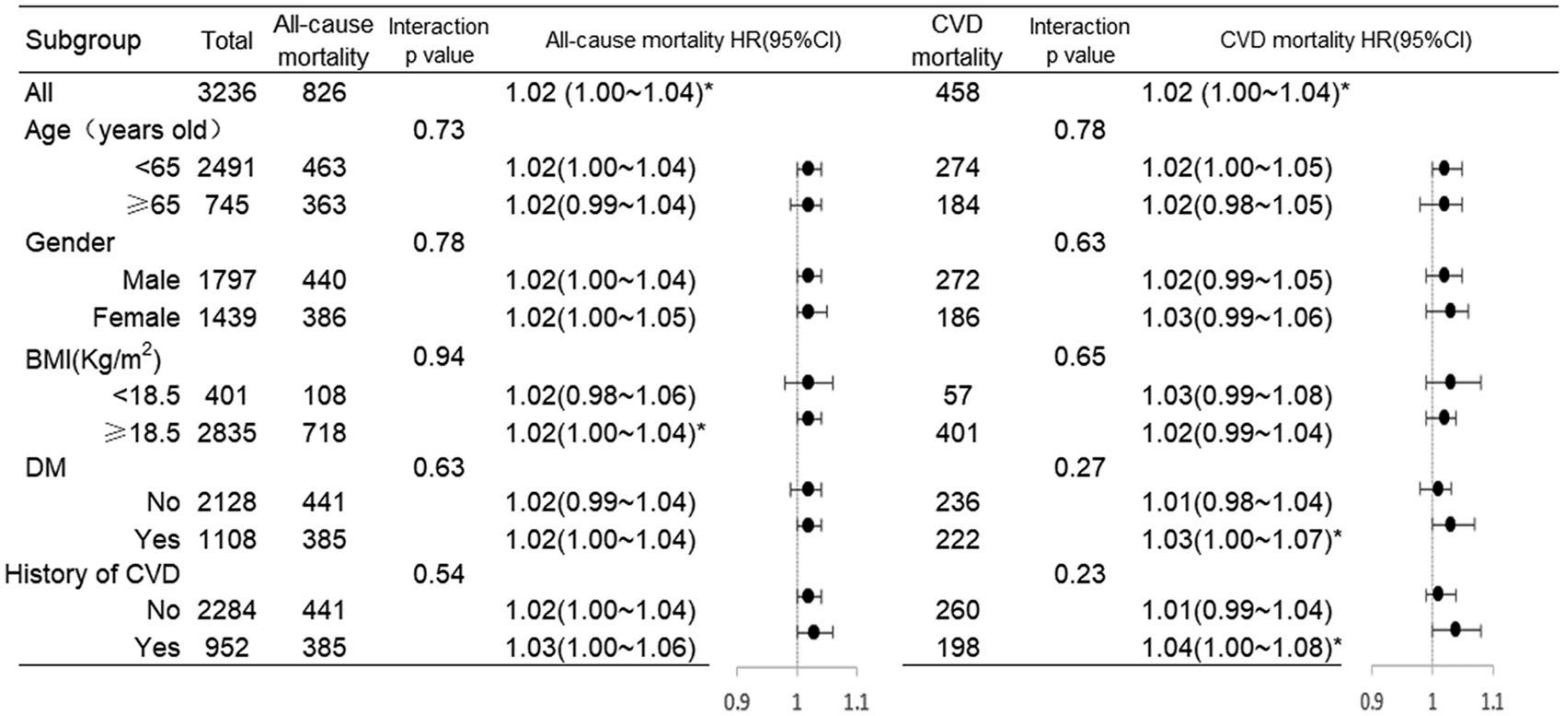
Forest Plot of the relationship between GLR and all-cause mortality in different subgroups. The interaction P value corresponds to the interaction test between the GLR and the subgroups variable of interest. No interaction was observed in terms of sex, age, BMI, DM and preexisting CVD in all-cause or CVD mortality. *p < .05.

We identified significant differences in all-cause and CVD mortality rates between the subgroups. Patients in the BMI ≥ 18.5 group had a higher risk of all-cause mortality (*p* = .04, HR 1.02; 95%CI 1.00 ∼ 1.04), while these trends were not observed in the BMI < 18.5 group. Further, patients with a higher risk of CVD mortality included those who had a history of DM (*p* = .03, HR 1.03; 95%CI 1.00 ∼ 1.07) or a history of CVD (*p* = .03, HR 1.04; 95%CI 1.00 ∼ 1.08). Moreover, no statistically significant interaction was observed for sex, age, BMI, DM, and CVD subgroups in all-cause mortality or CVD mortality (*p* > .05) (Figure 6).

## 4. Discussion

GLR is a newly described marker related to metabolic syndrome and chronic inflammation status, which assesses both fasting blood glucose and lymphocyte count, and indicates hyperglycemia and lymphocyte metabolism. In the present study, higher GLR levels were found to be associated with increased risks of all-cause mortality and CVD mortality due to metabolic derangement and chronic inflammatory response. Hyperglycemia may induce microenvironmental hypoxia [11], contribute to a significant increase in epidermal growth factor (EGF) transcription or secretion [12], activate chronic inflammation [13], and is associated with a higher risk of mortality in dialysis patients [2]. Oxidative stress driven by hyperglycemia leads to β-cell dysfunction, peripheral tissue insulin resistance, and new-onset diabetes [14], which are risk factors for cardiovascular events. Compared to the general population and patients with chronic kidney disease, patients undergoing PD have a higher risk of experiencing excessive oxidative stress (OS), which may exacerbate chronic inflammation, atherogenesis, and loss of residual renal function [15,16]. First, it has been proposed that the composition of PD fluids with low pH, elevated osmolality, and increased lactate concentration may promote the oxidation of peritoneal mesothelial cells [17]. Second, glucose degradation products, which come from the heat sterilization technology of PD fluids, enhance the accumulation of advanced glycation end-products and participate in long-term remodeling of the peritoneal membrane [18].

PD patients show unbalanced lymphocyte metabolism, which is normally tightly regulation by the immune response to glycolysis [19,20], and the expansion and function of T cells may be repressed under high glucose conditions. Malnutrition, accumulation of uremic toxins, and inflammation may lead to the inability to gain adequate nutrients, thereby accelerating the aging process of premature T cells, and causing significant barriers to T cell function in patients with PD [21]. Naïve T cells play a key role in the maintenance of adaptive immunity. Alterations in T cell subsets may attenuate the response to antigens and increase the risk of CVD in patients with kidney failure [22]. CVD mortality is influenced by a variety of factors, including smoking status, blood pressure, cholesterol, and overweight [23,24], while the importance of hyperglycemia and abnormal lymphocyte metabolism should not be ignored.

The mechanism underlying GLR involves inflammation in addition to abnormal glucose and lymphocyte metabolism. Chronic inflammation [25] causes dramatic structural and functional changes in the peritoneal membrane. The causes of chronic inflammation in PD patients include both dialysis-related (stimulation of peritoneal catheters, high-glucose, high-GDP dialysis solutions, complement activation, peritonitis, and exposure to endotoxins) and CKD-related (loss of residual kidney function, accumulation of uremic toxins, and chronic heart failure or fluid overload) [26]. Previous studies have found that some traditional inflammatory markers may further amplify long-term mortality and cardiovascular event risk in patients with PD [27,28].

As a novel marker of inflammation, GLR deserves more attention in PD patients. In this research, Spearman’s analyses showed that the GLR level was positively correlated with PLR and NLR, which may also reflect the chronic inflammation status of PD patients, leading to poor survival outcomes. it has been reported that NLR and PLR are clinically accepted as markers of systemic inflammatory response [29,30], as well as predictors of cardiovascular events and cancer. Furthermore, stratified analysis suggested that GLR was significantly associated with higher CVD mortality risk in patients who were overweight [31], had diabetes, and preceding CVD, which agrees with the results of previous studies [4,6]. This might indicate that PD patients with DM and preceding CVD tended to have multiple complications and shorter life expectancies, similar to aged participants. In this study, no interaction was observed for sex, age, BMI, diabetes mellitus, and preexisting cardiovascular disease in all-cause mortality or CVD mortality, indicating a stable association between GLR and poor prognosis.

Compared with traditional markers of the inflammatory response, GLR has better sensitivity and specificity than NLR, PLR, and LMR [8], which are easily available and cost-effective. In addition, there have been several prognostic analysis about the value of new inflammatory markers in PD patients, such as NLR [32] and an-immune-inflammation value (PIV) [33]. However, compared with the general population and patients with chronic kidney disease, patients undergoing PD have a higher risk of hyperglycemia. Related to metabolic syndrome and chronic inflammation status, GLR is more appropriate for PD patients than other markers of inflammation.

## 5. Limitations

Based on the retrospective design, this study has some inevitable limitations. Firstly, the causal association between GLR and mortality was unclear in observational study. Secondly, some variables, which have been found to be associated with increased CVD mortality risk, such as peritonitis or HbA1c value, were unavailable in our study. Also, dynamic changes in the GLR were not available during the follow-up period. Finally, some patients with diabetes mellitus may have taken antidiabetic drugs or injected insulin at the time of blood sample collection. Thus, a larger prospective study is required to validate our results.

## 6. Conclusion

In summary, our study demonstrated that increased serum GLR is an independent prognostic factor of all-cause and CVD mortality in patients with PD, but the underlying mechanisms are intricate and warrant further exploration.

## Data Availability

The datasets used are available from the corresponding author on reasonable request.
